# Controlling *Botrytis elliptica* Leaf Blight on Hybrid Lilies through the Application of Convergent Chemical X-ray Irradiation

**DOI:** 10.5423/PPJ.OA.09.2015.0187

**Published:** 2016-04-01

**Authors:** Sung-Jun Hong, Tae-Hoon Koo, Sung-Chul Yun

**Affiliations:** Department of Biomedical Sciences, Sun Moon University, Asan 31460, Korea

**Keywords:** NaDCC, phytotoxicity, vase life

## Abstract

X-ray irradiation with convergent chemicals such as nano-silver particles or sodium dichloroisocyanurate (NaDCC) has been used to control leaf blight on cut lilies. The oriental hybrid lily cultivars Siberia, Le Reve, and Sorbonne were irradiated five times by 200 Gy of X-rays in 2014. In 2015, Siberia and Sorbonne were irradiated three times by 150 Gy of X-rays. After artificial infection with *Botrytis elliptica* on the leaves and petals of cut lilies, this study used convergent chemical X-ray irradiation of 200 Gy or 150 Gy. Leaf and petal blight was measured in terms of incidence and severity at 8 days after infection using total 552 cuttings. Results indicate that the treatments of X-ray irradiation and NaDCC in 2014 and 2015 slightly decreased the severity of petal blight on Siberia and Sorbonne. However, the results were not significant and severity did not decrease as NaDCC concentration increased. Vase-life was observed separately after X-ray irradiation of 270 cut lilies in 2014 and 108 cut lilies in 2015. Chlorophyll content was not affected by either 200 Gy or 150 Gy of X-rays. The number of days of fully opened flowers at Siberia of 150 Gy and Le Revu of 200 Gy increased by 1–2 days. In addition, the relative fresh weights of the X-rayed flowers were 10% drier than the non-irradiated controls. Overall, leaf blight control by X-ray was inferior to the control by gamma rays, and petal color was bleached in Sorbonne and Le Reve cvs. by 150 Gy of X-rays.

Cut lilies are an important agricultural good in Korea; exports to Japan alone totaled more than $3,009,000 US. in 2012 (KITA, 2012). The main cultivars (cvs.) for export are the oriental hybrids, which are bigger and have a stronger scent (Taean LES, 2014). Leaf blight on these lilies is a serious disease, not only during cultivation in the field or greenhouse, but also during storage and transportation. The pathogens of this disease are *Botrytis elliptica* and *B. cinerea*, though the major threat is *B. elliptica* (Hahm et al., 2007; KSPP, 2009). Because disease control has been highly dependent on preventative fungicidal sprays, resistant-isolates of the pathogen have arisen against several fungicides, including benomyl and diethofencarb (Kim et al., 2001). Occasionally, spots of fungicide on leaves have also deteriorated lily quality and reduced export value.

Since methyl bromide is no longer allowed in quarantine, many alternatives have been suggested (Kwon et al., 1999). Ionizing radiation is one nonchemical treatment option for the control of postharvest diseases (Hallman, 2011). Ionizing radiation creates sufficient energy (via the interaction of atoms with damaged molecules) to produce free radicals that are highly active and can break chemical bonds without any residue. For pest control, three sources of ionizing radiation are commercially used: the gamma ray, X-ray, and electron-beam (De Silva et al., 2006). Gamma rays are the most popular and widely used method of control among these three sources. Moreover, gamma rays have been known to extend the vase life span of cut flowers (Park et al., 1999).

Compared to gamma irradiation, reports of X-ray use for the control of postharvest disease are very limited (Hallman, 2011). X-rays are a distinctly different source of ionizing radiation to gamma rays. Ionizing radiation shares a mode of action: that is sufficient radiation energy to render atoms and molecules ionized and excited. It creates free radicals, which can break chemical bonds and damage molecules in exposed cells without leaving any residue. Among the three commercially used ionizing radiation sources (gamma rays, X-rays, and electron-beams) gamma rays have been evaluated most thoroughly for use during the storage of pathogenic fungi. The degree of control of the storage fungi was found to be similar regardless of the ionizing source used (Jeong et al., 2015). According to earlier X-ray irradiation studies on tulips (Myodo, 1952), high-dose injuries have been observed, including decreased height, increased branching, irregularities in leaf shape, and delayed or reduced blossoming. The active plant organs were especially sensitive compared to the seeds and resting nutritive organs. As a result, when we apply X-ray irradiation to control disease, it is necessary to investigate cut flower quality after high-dose exposure, which includes examining features such as chlorophyll content, flower blossoming, and changes in fresh weight.

Ionization radiation is an eco-friendly method of insect control that does not leave any residue. Since more than 1 kGy of ionization radiation is required to control fungal disease on plants (Kader, 1986), most studies have investigated the use of convergent chemicals in combination with gamma rays, X-rays and e-beams (Jeong et al., 2015). Since these three types of ionization radiation have the same mode of action, and a similar dose-range of irradiation is used to reduce fungal survival and germination, this study aimed to evaluate X-rays as an alternative radiation source to gamma irradiation, which has been widely used and studied within our research group (Kim et al., 2014; Kim and Yun, 2014). Among the listed convergent chemicals, sodium dichloroisocyanurate (NaDCC) has been used safely in dental clinics since the 1980s and is a consistently stable disinfectant. Nano-silver particles (NSS) also have the potential to inhibit fungal germination and hyphal growth and the particles have been shown to effectively control cucumber powdery mildew (Park et al., 2006). Sodium hypochlorite (NaOCl) has been widely used as a disinfectant and is also potentially comparable with NaDCC.

Our research group’s previous gamma ray studies on the control of cut lily leaf blight suggest that 200 Gy of gamma rays with 40 μg/l of NaDCC in vitro would be effective as a pest control option (Kim and Yun, 2014). Gamma rays and NSS were slightly more effective than in the first in vivo study than gamma ray and NaDCC using cut lilies (Kim et al., 2014). To apply this technique to commercial exports, a safer and more secure way of controlling the blight without the side effects of ionization radiation is needed. In turn, it is critical to examine the potential of X-rays (and the previously successful convergent chemicals) and to compare its control efficacy with gamma rays options.

## Materials and Methods

### Lilies and the leaf blight pathogen

The lily cvs. used in this experiment were all oriental hybrids; the Siberia cv. is white in color, and is the most popular cv. Sorbonne and Le Reve are pink-colored varieties. They were harvested and cut to 90 cm in length 1 day before X-ray and chemical treatments. Each lily stem had three to five flowers, and the lowest flower had opened earlier than the others. When the cut lily stem was treated, even the lowest flower was green and had not yet opened. In a 2015 experiment, white (cv. Siberia) and colored (cv. Sorbonne) cvs. were used. The pathogen of the lily leaf blight was *Botrytis elliptica* (NO. 43461) and was taken from the Korean agricultural culture collection. To harvest conidial suspensions, *B. elliptica* was cultured on Potato Dextrose Agar (Difco, USA) at 22°C for 7 days under near ultra-violate light for twelve 12 h cycles. The harvested conidial suspensions for infection were adjusted to 5 × 10^5^ spores/ml.

### X-ray convergent treatments with several chemicals for control of lily leaf blight

After spraying the pathogen conidial suspensions on the cut lilies, the stems were put in a humid chamber controlled to about 95%–100% relative humidity at 19°C for 24 h to create a conducive environment. Then, the diseased cuttings were treated with the convergent chemicals and irradiated X-rays at 200 Gy in 2014 and 150 Gy in 2015. In 2014, the three chemicals selected as treatments were NaDCC 40 μg/l, NSS 40 μg/l, and NaOCl 800 μg/l, and the cuttings were sprayed until the chemicals ran-off. The treated cuttings were wrapped in a carton box for export (20 cm (W) × 100 cm (L) × 15 cm (H)) and transported to X-ray facilities at EB-tech (Daejeon, Korea). When X-ray irradiation occurred, most of the lily cuttings did not yet have open flowers. Irradiation was conducted a total of five times, once on each of the following days: 11^th^ February, 18^th^ February, 26^th^ February, 5^th^ March, and 7^th^ March, 2014. Each time, the X-ray irradiation was a replication. The eight treatments used a combination of X-ray irradiation (0 Gy and 200 Gy) and the four convergent chemicals, including water, as a control. In summary, a total of eight treatments were used: 1) 0 Gy + water; 2) 200 Gy + water; 3) 0 Gy + NaDCC 40 μg/l; 4) 200 Gy + NaDCC 40 μg/l; 5) 0 Gy + NSS 40 μg/l; 6) 200 Gy + NSS 40 μg/l; 7) 0 Gy + NaOCl 800 μg/l; and 8) 200 Gy + NaOCl 800 μg/l. Three cuttings were used for each treatment, giving a total of 24 cuttings of each cv. In addition, 18 cuttings per cv. (nine irradiated + nine non-irradiated) were used for the vase life experiment. A total of 42 cuttings per cv. were used in each irradiation; 126 cuttings for each of the three cvs. × 5 irradiation treatments = 630 cuttings total used in the 2014 experiment. After X-ray irradiation, the treated cuttings were re-cut in water 15 cm from the tip and put in a 2 l Erlenmeyer flask to measure disease characteristics and conduct the vase life study. This study occurred in room conditions under florescent light for 14 days.

From a 2015 study, we knew that 200 Gy of X-rays was slightly harmful to colored cvs. and as a result decided to decrease irradiation levels to 150 Gy. In addition, NaDCC was the most effective of the convergent chemicals. Therefore, several concentrations of NaDCC were used to find an optimal concentration. The eight treatments chosen were the products of X-ray irradiation (0 Gy and 150 Gy) and the four levels of NADCC (0 μg/l, 40 μg/l, 100 μg/l and 200 μg/l). The non-irradiated (0 Gy) treatment with water sprayed (0 μg/l) acted as a control. Only two cvs. were used, one white (cv. Siberia) and the other colored (cv. Sorbonne). X-ray irradiations at 150 Gy took place at Seoul Radiology Services (Eumseong-gun, Chungbuk-do Korea). Irradiation was conducted a total of three times: on 27^th^ February, 10^th^ March, and 13^th^ March, 2015. In each irradiation replication, there were eight treatments and 4 cuttings were used. Since cvs. Siberia and Sorbonne were used, total 64 cuttings for the disease experiment. In addition, 18 cuttings per cv. (nine irradiated + nine non-irradiated) were used for the vase life experiment. Since cvs. Siberia and Sorbonne were used, a total of 64 cuttings for the disease experiment and 36 cuttings for vase life study per cultivar were used. Total 100 cuttings for each irradiation replication were used total 300 cuttings were used in the 2015 study. After X-ray irradiation, the treated cuttings were re-cut in water 15 cm from the tip and put in a 2 l Erlenmeyer flask. They were then measured for disease characteristics and used for the vase life study in room conditions for 14 days.

### Vase life measurement for X-ray irradiation at 200 and 150 Gy

Vase life is essential to maintain freshness and was measured in accordance with three characteristics: 1) chlorophyll content; 2) fresh weight; and 3) days of fully opened flowers, either lasting 2 weeks or until they wilted. Chlorophyll content was measured with a SPAD chlorophyll meter (SPAD-502, Konica Minolta, Tokyo, Japan) on the first and twelfth day after irradiation to check the effects of irradiation, and for changes during the elapsed time. The fresh weight of the cuttings was measured on the first, fifth, and twelfth day after irradiation. Relative fresh weight was calculated as 100% of the 1-day weight of the cutting to check for loss of moisture depending on the irradiation level and elapsed time. The duration of the fully opened flower was measured using the three flowers closest to the bottom of the cutting (i.e., the lowest, low, and middle flowers) and was taken as the time between the flower being fully opened until it wilted. In most cuttings, the lowest flower opened earlier and stayed open longer than the others.

### Measurement of disease (incidence and severity)

Leaf blight symptoms were visible on the leaves and on the petals after the infection was applied. Incidence and severity were measured in each treated cutting. The symptoms on the leaves and petals were measured 8 days after inoculation. Disease incidence on the leaves was measured according to whether the inoculated cutting was with or without infected leaves (eight leaves per cutting). Therefore, incidence was represented by the percent of infected leaves out of 24 leaves, because three cuttings were used in each treatment. If one or two leaves had fallen during the experiment, approximately six to eight leaves were measured. Disease severity on the leaf was categorized using a symptom appearance scale ranging from 0 to 4 (level 0, no symptoms; level 1, 1–10% symptoms; level 2, 11–25% symptoms; level 3, 26–50% symptoms; and level 4, > 50% of the overall area was symptomatic). The severity of the numerator was the sum of the symptom scores of the eight leaves. The severity of denominator was 4 (the maximum level) × 8 leaves. A percentage was then calculated for disease incidence. Since four cuttings were used in 2015, the disease incidence and severity should be considered for four cuttings.

$$\begin{array}{l} \text{Incidence }(\%)=\frac{\text{number of diseased leaves}}{24\;\text{leaves }(8\;\text{leaves}\times 3\;\text{cuttings})}\times 100\\ \text{Severity }(\%)=\frac{\text{Sum of the levels of }8\;\text{leaves}}{96\;(24\;\text{leaves}\times 4\;(\text{max.}\;\text{level})}\times 100 \end{array}$$

Disease incidence on the petals was similar to that on the leaves. We measured three flowers per cutting and three petals per flower (3 flowers × 3 cuttings = 9 petals per treatment) and the flowers were not yet open. Disease severity was characterized in the same manner for petals as for leaves:

$$\begin{array}{l} \text{Incidence }(\%)=\frac{\text{number of diseased leaves}}{27\;\text{petals }(9\;\text{petals}\times 3\;\text{cuttings})}\times 100\\ \text{Severity }(\%)=\frac{\text{Sum of the levels of }9\;\text{petals}}{108\;(27\;\text{leaves}\times 4\;(\text{max.}\;\text{level})}\times 100 \end{array}$$

After the measured flowers were fully open, we measured six petals per flower instead of three.

### Statistical analyses

Statistical comparisons were performed for disease incidence and severity among the eight treatments in each cv. Chlorophyll content was compared among four different irradiation (X-ray, irradiated and non-irradiated) and measurement time (1 and 12 days after irradiation [DAI]) combinations, for each cv. One-way analysis of variance and further comparisons using least significant difference (LSD) tests were conducted using the S-Link software (S-Link, Seoul, Korea). The duration of fully opened flowers was compared among X-ray irradiation treatments for each flower position (i.e., for the lowest/low/middle flowers in a cut lily) for every cv. Relative fresh weight was compared only on the twelfth day after irradiation between the irradiated and non-irradiated treatments. Student’s *t*-test was performed using S-Link.

## Results

### X-ray and convergent chemical effects on petal blight

Blight on the petals was more frequent and severe than on the leaves, especially in the 2015 experiment. Table 1 shows the disease incidence and severity on petals. Although 2-year experiments were conducted using the same cvs. and isolates of the pathogen, disease symptoms were much more severe in 2014 than in 2015. Siberia and Sorbonne cvs. in 2014 showed around 70% disease incidence and 35% severity, whereas Le Reve showed 60% incidence and 25% severity. In 2015, disease incidence and severity rates on Sorbonne were 55% and 14%, which were slightly higher than those of Siberia. Disease incidence and severity did not differ among cvs. treated using the eight X-ray plus chemicals treatment combination, for either year. Neither X-ray treatments (200 Gy or 150 Gy) nor several convergent chemicals, including NaDCC, improved disease control. By comparing 0 Gy and 200 Gy, or 150 Gy and control, we can assess whether X-ray irradiation was able to control disease without chemical assistance. Decreased disease incidence rates using 200 Gy of X-ray were found on Le Reve and Siberia at 10% and 5%, but this was not found on Siberia. In addition, 150 Gy of X-ray did not decrease disease incidence on Siberia or Sorbonne (Table 1). Decreases in disease severity of 3–4%, using X-ray irradiation in both years, were found on Siberia but not on the other cvs. Among the three convergent chemicals, NaOCl and NaDCC were slightly more effective than NSS, which was a nanoparticle. In the 2015 experiment, with 40 μg/l, 100 μg/l, and 200 μg/l of NaDCC treatment, 40 μg/l slightly decreased disease incidence, to 7% and 6%, and decreased disease severity, to 2% and 1.3%, on Siberia and Sorbonne, respectively (without using X-ray irradiation). Furthermore, 150 Gy of X-ray irradiation and 40 μg/l NaDCC decreased disease incidence to 8.7% and 8.5%, and severity to 4.3% and 0.8%, on Siberia and Sorbonne, respectively. However, synergy effects were not found using 100 μg/l of NaDCC; those treatments slightly improved at 200 μg/l (shown in Table 1). It remains unclear whether disease control can be improved by changing NaDCC concentrations.

### X-ray and convergent chemical effects on leaf blight

Blight rates for the lily leaves are listed in Table 2 for the 2014 experiment only, as disease on the leaves in the 2015 experiment was negligible. Unlike on the petals, disease incidence and severity rates were highest for Le Reve cv. at 46%–62%, compared to 14%–22% among the three other cvs. However, the difference among the other cvs. was not significant. Statistical comparisons of the eight different combinations of X-rays and the three chemicals in each cv. showed no significant differences in disease incidence or severity control. Neither 200 Gy of X-ray irradiation nor the use of three convergent chemicals improved disease control (Table 2). Slight decreases in disease incidence and severity, of 4% and 1.4%, respectively, were only found in the Siberia cv. and this was not associated with increased NaDCC concentrations, but rather with irradiating X-rays. The three convergent chemicals were not an effective means of decreasing disease symptoms on the leaves of the three cvs.

### Vase life changes

**Fig. 1:**
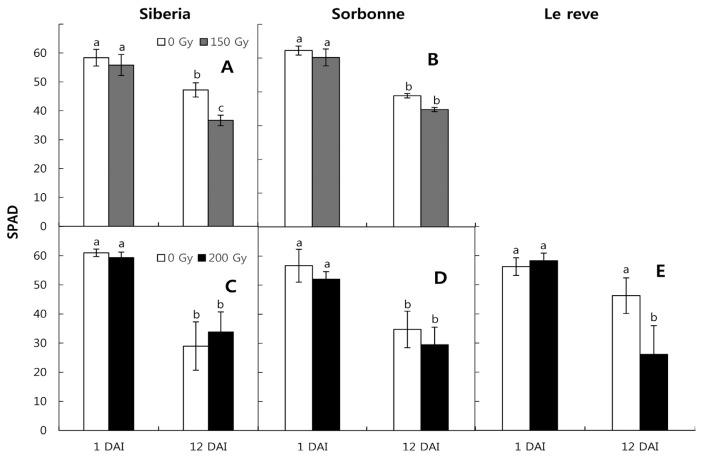
Chlorophyll contents of the lily leaves of three oriental lily cvs.: Siberia (A, C); Sorbonne (B, D); and Le Reve (E). Chlorophyll contents were measured by SPAD on nine leaves treated by gamma irradiation (0 (□) and 150 (■) Gy; A, B; and 0 (□) and 200 (■) Gy; C, D, E) immediately and 12 days after irradiation (DAI). Values are shown as means ± SE of three replications, which were independently irradiated. In each cv., statistical comparisons were conducted among four different treatments, (i.e., different combinations of gamma irradiation intensities (0 and 200 Gy or 0 and 150 Gy) and dates of measurement (immediately and 12 days after irradiation).

To find the X-ray effect on the vase life of lily cuttings, we measured the chlorophyll content of leaves, fresh weight changes, and duration of fully opened flowers. Chlorophyll content was slightly decreased immediately after irradiation on day 1, except for in the Le Reve cv., but not significantly (Fig. 1). In comparison, relative to day 1 at 0 Gy, chlorophyll was significantly decreased after 12 days, except for in the Le Reve cv. For 0 Gy and 150 Gy or 200 Gy at day 12, the X-ray irradiated plants was slightly lower in chlorophyll content, but there were no significant changes except for in the Le Reve cv. (Fig. 1).

**Fig. 2:**
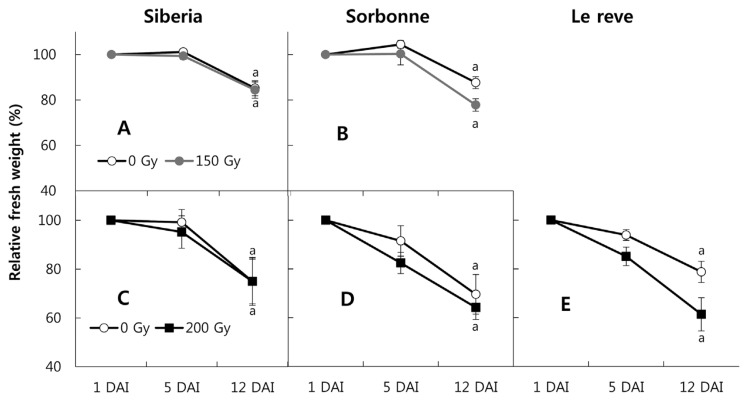
Changes in relative biomass (from 100%) were measured at 1, 5, and 12 days after irradiation (DAI). Doses of X-ray irradiation were 0 (□) and 150 (■) Gy (A, B) and 0 (□) and 200 (■) Gy (C, D, E). The three oriental cvs. were: Siberia (A, C); Sorbonne (B, D); and Le Reve (E). X-ray irradiation was conducted independently three times. *T*-test comparisons were done between X-ray-irradiated and non-irradiated cuttings 12 DAI in each cv.

The change in the relative fresh weights of lily cuttings may be associated with water use and the significant decreases are likely to be related to wilting or drying. For the Siberia cv., fresh weights decreased to 70%–80%, but 150 Gy and 200 Gy of X-ray irradiation did not affect the loss of fresh weight (Fig. 2). For the Sorbonne cv., X-ray irradiation slightly accelerated the fresh weight loss compared to the control, but not significantly (Fig. 2B–D). Differences among the X-ray treatments were found for the Le Reve cv., starting from day 5 after x-ray irradiation at 200 Gy, which became much more pronounced at day 12 (Fig. 2E). Relative fresh weight loss by X-ray irradiation on the Le Reve cv. was 70%, which was similar to the Siberia and Sorbonne cvs. However, the relative fresh weight of Le Revu maintained at 80%, which was higher than for both the Siberia and Sorbonne cvs. (Fig. 2).

Typically, the lily cuttings had three to five flowers; the lowest one opened first and remained open longer than the others. In the 2014 experiment, flowers in the lowest and low positions in the cuttings of the three cvs. lasted for 4–6 days, whereas those in the middle position lasted for 3–4 days. By using 200 Gy of X-ray irradiation, fully open flowers were maintained at the same position for less than 1 day (Table 3). Using 150 Gy of X-ray maintained the lowest and low flowers on the Siberia cv. for 1–2 days, but they were not maintained on the Sorbonne cv.

## Discussion

Although previous gamma experiments showed that NaDCC was the best and most promising convergent chemical (Kim and Yun, 2014), neither X-ray irradiation using 200 Gy nor the convergent chemicals with X-ray irradiation were effective at controlling lily blight for the purposes of application to commercial fields. It is also necessary to examine flower quality if applying X-ray irradiation of 1–2 kGy, as high levels of ionizing irradiation can cause blackening at the flower tip, failure of flower opening, accelerated dryness, bleached petals, and degrading chlorophyll (Myodo, 1952). Considering that at least 1–2 kGy X-ray irradiation is needed to eradicate fungal pathogens (Kader, 1986), relatively high doses of the chemical convergent is similarly needed to control leaf blight without phytotoxicity effects. Even at 200 Gy of X-ray or gamma ray irradiation, phytotoxicity was occasionally found on a cut flower’s tender tissues, such as the petals. In this regard, 150 Gy was considered to result in better flower quality than 200 Gy.

This leaf blight experiment aimed to provide curative control using X-ray irradiation only, chemicals only, or a combination of the two techniques. We assumed that *B. elliptica* causes infections in the field or during storage (including transportation) and that the symptoms are revealed once the lilies are on the market. Thus, the best time to control blight with X-ray irradiation is during the export and quarantine processes. Even commercial fungicides used as curative controls (which inhibit mycelial growth) are much more difficult to apply than preventive controls, which inhibit spore germination. Because high doses of ionizing irradiation, including X-rays, damage fungal spores and inhibit spore germination (Jeong et al., 2015), X-rays may have curative as well as preventive potential for controlling fungal diseases during the infection process. However, a practical low dose, such as 150 or 200 Gy, of X-rays did not achieve the expected curative control through damage of the pathogen spores. The results of this study did not indicate decreased disease incidence or severity as a result of the X-ray treatment. However, the same levels of gamma rays have shown 10–20% decreases in disease incidence regardless of the NaDCC concentrations applied (data not shown).

We had some promising results with X-ray treatments. X-rays at 150 Gy maintained 1 to 2 days of bloom opening for the low and lowest flowers on Siberia cvs. The same intensity of gamma rays at best added 0.5 to 1 additional days of flower opening (Kim et al., 2014). Park et al. (1999) reported longer vase life in cut roses using gamma rays. In this study, X-rays gave longer vase life to opened flowers and was slightly better than gamma irradiation. In addition, the fresh weights of cut lilies that were X-ray irradiated were maintained up to 12 DAI, similar to those of non-irradiated cuttings. Those that underwent gamma irradiation had significantly decreased fresh weights (Kim et al., 2014). Water absorption was prevented by the physiological malfunction of the harvested cut flowers and accelerated dryness was found with 200 Gy of gamma irradiation (Kim et al., 2014). It appeared that X-rays did not affect the post-harvest physiology of cut lilies because their fresh weight was maintained. Longer leaf freshness in a vase is another indicator of cut flower quality. Additionally, 500–750 Gy of gamma irradiation destroyed chlorophyll content (Kang et al., 2004). In this study, 150 or 200 Gy of X-ray irradiation did not immediately break down chlorophyll, as was the case for gamma rays. Chlorophyll loss was mainly spontaneous, as was longer vase life.

Bleaching of the colored petals by X-ray on Sorbonne and Le Reve cvs. (which are pink) was another concern with low dose treatments. Pale petal color was exacerbated by X-ray irradiation. Bleaching was more severe with 200 Gy of X-rays than with the same intensity of gamma rays. Even at 150 Gy of X-rays, bleached petals were found (data not shown).

In conclusion, we recommend applying gamma irradiation to commercial exports. Gamma rays had better leaf blight control, and markedly fewer phytotoxic effects, than X-rays. Siberia was the most insensitive cv. to X-rays and gamma rays under 200 Gy. Since it has white petals, it is not necessary to be wary of bleaching. Siberia cvs. also maintained fresh weight longer than any other cv. Moreover, its fully opened flowers remained open for 1–2 days longer.

Even though this study and others on cut lilies may not be sufficient to justify irradiation as a control for leaf blight during the quarantine and export processes, it is still worth investigating ionization irradiation as an alternative to methyl bromide. Because X-ray and gamma irradiation has a broad spectrum of activity against pests, insects and bacteria are much more sensitive to irradiation energy than fungal pathogens. D_10_ values for fungal plant pathogens are much higher than for insects and bacteria, and convergent chemicals are definitely needed to control plant pathogenic fungi by ionization irradiation.

## Figures and Tables

**Table 1:** Incidence and severity of oriental lily blight on petals. Artificial infection with conidial suspension of *Botrytis elliptica* on cut lilies 1 day before X-ray irradiation at 200 Gy in 2014, with several chemical convergents, and at 150 Gy in 2015, with various sodium dichloroisocyanurate concentrations. Data are provided as means ± SE of five (2014) and three (2015) replications of independent irradiation. Statistical comparisons among eight treatments (irradiation × chemicals) are shown for each cultivar

Experiment	Cultivars	Chemical Convergent	Incidence^1^		Severity^2^	
	
			0 Gy	200 Gy	0 Gy	200 Gy
2014	Siberia	Control^3^	72.9±6.8 ^a^	67.1±5.2 ^a^	31.0±4.8 ^a^	27.6±4.4 ^a^
		NaDCC (40)^4^	73.0±9.8 ^a^	67.3±7.3 ^a^	32.2±7.2 ^a^	26.4±4.0 ^a^
		NSS (40)	79.7±6.8 ^a^	75.9±3.3 ^a^	31.4±5.6 ^a^	34.8±2.8 ^a^
		NaOCl (800)	62.1±8.1 ^a^	73.2±11.3 ^a^	25.2±5.6 ^a^	29.3±6.0 ^a^

	Sorbonne	Control	69.7±8.3 ^a^	70.9±6.1 ^a^	35.6±6.6 ^a^	36.4±5.2 ^a^
		NaDCC (40)	63.3±9.9 ^a^	63.3±10.5 ^a^	29.1±5.4 ^a^	28.4±6.2 ^a^
		NSS (40)	69.2±9.4 ^a^	65.5±9.9 ^a^	32.4±7.7 ^a^	31.8±5.9 ^a^
		NaOCl (800)	74.4±10.5 ^a^	80.0±7.9 ^a^	33.8±7.0 ^a^	37.6±6.6 ^a^

	Le reve	Control	61.1±6.9 ^a^	48.9±14.6 ^a^	25.4±5.4 ^a^	24.6±8.0 ^a^
		NaDCC (40)	55.3±10.7 ^a^	48.4±10.5 ^a^	26.5±9.1 ^a^	22.1±5.4 ^a^
		NSS (40)	55.0±9.4 ^a^	56.9±9.9 ^a^	24.4±8.5 ^a^	26.9±6.0 ^a^
		NaOCl (800)	52.9±10.5 ^a^	46.4±7.9 ^a^	22.15±8.9 ^a^	21.5±6.4 ^a^

		NaDCC	0 Gy	150 Gy	0 Gy	150 Gy

2015	Siberia	Control	34.8±11.8 ^a^	37.7±14.7 ^a^	15.35±6.5 ^a^	10.9±5.2 ^a^
		40 μg/l	28.1±16.2 ^a^	28.9±5.2 ^a^	13.28±7.6 ^a^	6.6±2.6 ^a^
		100 μg/l	46.0±10.4 ^a^	47.0±9.0 ^a^	16.91±5.8 ^a^	9.8±3.4 ^a^
		200 μg/l	29.9±6.8 ^a^	34.7±3.6 ^a^	12.55±4.3 ^a^	8.1±1.7 ^a^

	Sorbonne	Control	54.5±6.8 ^a^	61.2±6.5 ^a^	13.9±2.9 ^a^	14.9±0.6 ^a^
		40 μg/l	49.1±10.7 ^a^	52.7±1.0 ^a^	12.6±0.6 ^a^	14.1±2.8 ^a^
		100 μg/l	65.3±8.4 ^a^	50.8±4.8 ^a^	16.3±4.9 ^a^	12.2±1.7 ^a^
		200 μg/l	55.1±10.0 ^a^	45.6±1.8 ^a^	13.9±3.4 ^a^	11.1±1.8 ^a^

1 Incidence was calculated as the percent of infected petals among three to four cuttings of lily.

2 Severity was the average level (0–4) of infection in the petals of each infected lily.

3 Control groups were treated with distilled water.

4 The numbers in parentheses are the concentrations (μg/l) of the chemicals.

**Table 2:** Incidence and severity of oriental lily blight on leaves. Artificial infection with conidial suspension of *Botrytis elliptica* on cut lilies 1 day before X-ray irradiation at 200 Gy in 2014 with several chemical convergents. Data are shown as means ± SE of five replications of independent irradiation. Statistical comparisons are among eight treatments (irradiation × chemicals) in each cultivar

Experiment	Cultivars	Chemical Convergent	Incidence^1^		Severity^2^	
	
			0 Gy	200 Gy	0 Gy	200 Gy
2014	Siberia	Control^3^	43.9±10.0 ^a^	39.8±7.5 ^a^	13.9±3.2 ^a^	12.5±3.0 ^a^
		NaDCC (40)^4^	42.6±9.5 ^a^	49.6±7.1 ^a^	12.2±3.3 ^a^	15.8±3.4 ^a^
		NSS (40)	40.2±8.6 ^a^	50.5±9.1 ^a^	13.5±2.6 ^a^	17.9±4.3 ^a^
		NaOCl (800)	51.3±5.2 ^a^	44.5±3.5 ^a^	16.8±1.8 ^a^	14.3±1.7 ^a^

	Sorbonne	Control	30.8±7.3 ^a^	35.0±6.8 ^a^	9.8±2.7 ^a^	11.9±2.5 ^a^
		NaDCC (40)	36.7±8.4 ^a^	35.5±7.7 ^a^	11.3±2.6 ^a^	10.1±2.6 ^a^
		NSS (40)	39.1±9.4 ^a^	42.9±10.1 ^a^	12.7±3.1 ^a^	13.1±3.8 ^a^
		NaOCl (800)	37.7±8.9 ^a^	41.7±7.0 ^a^	11.5±2.9 ^a^	12.9±2.4 ^a^

	Le reve	Control	46.5±2.2 ^a^	53.9±5.9 ^a^	14.0±0.5 ^a^	16.6±2.7 ^a^
		NaDCC (40)	47.7±4.0 ^a^	53.8±8.1 ^a^	15.2±1.3 ^a^	17.0±4.6 ^a^
		NSS (40)	51.1±7.6 ^a^	62.3±7.1 ^a^	17.5±3.3 ^a^	22.1±2.7 ^a^
		NaOCl (800)	54.9±5.8 ^a^	50.1±2.4 ^a^	18.2±1.8 ^a^	15.9±0.9 ^a^

1 Incidence was calculated as the percent of infected petals among three cuts of lily.

2 Severity was the average level (0–4) of infected petals of each infected lily.

3 Control groups were treated with distilled water.

4 The numbers in parentheses are the concentrations (μg/l) of the chemicals.

**Table 3:** Number of days of fully opened flowers for the oriental lily. After X-ray irradiation at 200 Gy (2014) and 150 Gy (2015), the treated cuttings were re-cut and reserved in vases. The number of days of fully opened flowers was measured as the time between being fully opened on day 1 and wilting. Statistical comparison between X-ray-irradiated and non-irradiated cuttings was done using *t*-tests for each flower position and cultivar

Oriental lily cultivar	Flower position	Days of fully open flowers			

		2014		2015	
	
		0 Gy	200 Gy	0 Gy	150 Gy
Siberia	Lowest	5.1±0.6 ^a^	4.8±0.8 ^a^	7.8±1.0 ^a^	9.1±0.9 ^a^
	Low	5.2±0.3 ^a^	4.3±0.4 ^a^	7.5±0.7 ^a^	9.5±0.4 ^a^
	Middle	3.1±0.5 ^a^	3.6±0.7 ^a^	5.3±2.3 ^a^	5.4±0.7 ^a^

Sorbonne	Lowest	5.9±0.6 ^a^	5.8±0.9 ^a^	10.0±0.3 ^a^	9.5±1.3 ^a^
	Low	4.8±0.4 ^a^	4.7±0.7 ^a^	6.2±0.7 ^a^	6.6±0.8 ^a^
	Middle	3.1±0.7 ^a^	3.3±0.8 ^a^	2.1±0.7 ^a^	2.1±0.9 ^a^

Le reve	Lowest	5.2±0.2 ^a^	4.5±0.7 ^a^	–	–
	Low	4.6±0.4 ^a^	4.3±0.5 ^a^	–	–
	Middle	2.9±0.4 ^b^	4.5±0.1 ^a^	–	–

## References

[b1-ppj-32-077] De Silva M, Moraes AML, Nishikwa MM, Gahi MJA, Vallim da Alencar MA, Brando LE, Nobrega A (2006). Inactiviation of fungi from deteriorated paper materials by radiation. Int Biodeter Biodegr.

[b2-ppj-32-077] Hahm SS, Lee KH, Lee JW, Lee HD, Yu SH (2007). Control and incidence of leaf blight on lily with different cultural systems. Res Plant Dis.

[b3-ppj-32-077] Hallman GT (2011). Phytosanitary applications of irradiation. Compr Rev Food Sci F.

[b4-ppj-32-077] Jeong R-D, Shin E-J, Chu E-H, Park H-J (2015). Effects of ionizing radiation on postharvest fungal pathogens. Plant Pathol J.

[b5-ppj-32-077] Kader AA (1986). Potential applications of ionizing radiation in postharvest handling of fresh fruits and vegetables. J Food Technol.

[b6-ppj-32-077] Kang GH, Seo JK, Ahn CH, Lee KS, Lee IS, Lee YI (2004). Chlorophyll mutation of radish irradiated with gamma ray. Kor J Hort Sci Technol.

[b7-ppj-32-077] Kim B-S, Chun HH, Hwang YA (2001). Occurrence and changes of Botrytis elliptica resistant to fungicides. Kor J Pesticide Sci.

[b8-ppj-32-077] Kim J-H, Yun S-C (2014). Effect of gamma irradiation and its convergent treatments on lily leaf blight pathogen, Botrytis elliptica, and the disease development. Res Plant Dis.

[b9-ppj-32-077] Kim J-H, Koo T-H, Hong S-J, Yun S-C (2014). Application of gamma irradiation and its convergent treatments on several varieties of oriental hybrid lily to control leaf blight. Res Plant Dis.

[b10-ppj-32-077] Korea International Trade Association (KITA) http://www.kita.net.

[b11-ppj-32-077] Korean Society of Plant Pathology (KSPP) (2009). List of plant disease in Korea.

[b12-ppj-32-077] Kwon HJ, Hwang MJ, Kim KS (1999). Postharvest physiology and prolonging vase life of cut freesia (Freesia refracta). Kor J Hort Sci Technol.

[b13-ppj-32-077] Myodo H (1952). Effects of x-rays upon tulip plants when irradiated in different developmental stages of floral organs. Journal of Faculty Agricultural Hokkaido Imperial University.

[b14-ppj-32-077] Park H-J, Kim SH, Kim HJ, Choi S-H (2006). A new composition of nanosized silica-silver for control of various plant diseases. Plant Pathol J.

[b15-ppj-32-077] Park I-H, Jung Y-S, Lee W-S, Kwon J-H, Byun M-W (1999). Effect of gamma irradiation and post-irradiation treatment of preservatives on the cut flower longevity of rose and mum. Kor J Postharvest Sci Technol.

[b16-ppj-32-077] Taean Lily Experiment Station (2014). http://lily.cnnongup.net/html/lily/.

